# Multimodal animal health monitoring in extensive livestock production systems

**DOI:** 10.3389/fvets.2026.1832869

**Published:** 2026-05-25

**Authors:** Xintao Zhao, Qiyu Liao, Dadong Wang, Erik Meijering, Cara Brosnahan, Suzanne Keeling, Rugang Tian, Wenrong Li, Mhairi Sutherland, Jie Kang

**Affiliations:** 1 School of Computer Science and Engineering, The University of New South Wales, Sydney, NSW, Australia; 2 CSIRO Data61, Sydney, NSW, Australia; 3 Beef + Lamb New Zealand, Wellington, New Zealand; 4 Inner Mongolia Academy of Agricultural and Animal Husbandry Sciences, Hohhot, China; 5 Institute of Animal Biotechnology, Xinjiang Uyghur Autonomous Region Academy of Animal Science, Urumqi, Xinjiang, China; 6 Sydney Precision Data Science Centre, The University of Sydney, Sydney, NSW, Australia; 7 Charles Perkins Centre, The University of Sydney, Sydney, NSW, Australia; 8 School of Mathematics and Statistics, The University of Sydney, Sydney, NSW, Australia

**Keywords:** animal health and welfare, disease surveillance, extensive livestock systems, multimodal monitoring, precision livestock farming

## Abstract

Animal production in extensive livestock systems faces significant health and welfare challenges due to variable environments, diverse climatic conditions, and practical constraints that limit close animal monitoring. By “extensive livestock systems”, we refer to production systems characterized by large herd sizes, open-range grazing, and limited direct animal supervision, typical of beef cattle, sheep, and goat farming in rangeland environments. Conventional approaches, including visual inspection and periodic veterinary assessment, often provide incomplete and delayed insights into animal health status, limiting timely intervention for infectious and metabolic diseases. Recent advances in wearable sensors, imaging technologies, genomic testing, omics profiling, and environmental monitoring offer new opportunities for continuous, data-driven surveillance of livestock. However, when applied in isolation, these modalities capture only partial aspects of the complex biological and environmental processes that influence animal health and disease progression. Multimodal monitoring integrates these diverse data streams to provide a more comprehensive and dynamic representation of animal health. This enables earlier detection of disease risk, improved welfare outcomes, and enhanced support for veterinary and on-farm decision-making. Ultimately, such integration empowers farmers to achieve earlier and more precise interventions, reduce veterinary costs, and improve overall animal welfare and productivity in extensive systems. This review synthesizes current approaches to multimodal monitoring in extensive livestock systems, explores data integration strategies, and evaluates key challenges for practical implementation, including cost, scalability, and data interoperability. We conclude by outlining future research directions that prioritize feasibility, affordability, and farmer-centered design to facilitate real-world adoption.

## 1 Introduction

Extensive livestock systems dominate animal production worldwide (1), particularly ruminant livestock, such as cattle, sheep, and goats. These systems are characterized by large herd sizes, open grazing environments, and limited direct handling and monitoring of animals. While such systems benefit from reduced labor and infrastructure costs, they simultaneously present major challenges for animal health and welfare due to limited direct observation and delayed detection of illness (2–5). Early signs of disease or distress may go unnoticed for days or weeks, delaying treatment, reducing treatment effectiveness, increasing welfare risks, and resulting in economic losses.

New technologies provide an opportunity to transform animal health monitoring in extensive systems (6–9). Wearables (10, 11), imaging (12), genomic selection (13, 14), omics profiling (15, 16), and environmental sensors (17) each capture different dimensions of animal health and performance. However, most current frameworks treat these modalities independently, limiting their value and potential. Integration across modalities promises more effective and practical monitoring solutions (18).

This review examines the role and potential of multimodal monitoring in extensive livestock systems for the purpose of real-time health management of direct benefit to farmers and veterinarians. It focuses specifically on opportunities, integration strategies, limitations, and pathways to adoption. It is important to acknowledge that a substantial proportion of current evidence for precision livestock farming and multimodal monitoring has been generated in intensive, confined dairy systems in temperate regions. In these settings, established infrastructure, reliable connectivity, and higher economic value per animal facilitate technology deployment and adoption (19). Adapting these findings to extensive livestock systems introduces several distinct challenges that are insufficiently addressed in the existing literature.

First, environmental heterogeneity in rangeland systems is substantially greater than in indoor and semi-enclosed environments. Variation in terrain, vegetation, and local environmental conditions may affect sensor performance and introduce potential confounders not encountered in controlled settings (20). Second, reliable network coverage is often absent in remote pastoral areas, constraining real-time data transfer and necessitating on-device processing or delayed upload architectures. Third, lower profit margins per animal in extensive systems limit adoption for expensive monitoring systems. Technologies validated in dairy herds may therefore be less economically viable for extensive livestock systems where cost and scalability constraints are more pronounced (6, 21). Fourth, the relative importance of different health risks varies between systems, with parasitic and nutritional challenges being more prominent in extensive grazing contexts (21). Finally, these differences have direct implications for the choice of sensor, data integration architecture, and validation protocol design.

Validation protocols must account for the higher environmental and behavioral variability inherent in extensive systems. Standard random split protocols developed in animal behavior classification contexts are unlikely to generalize without adaptation (22). A growing body of work has begun to address some of these challenges. Studies of Merino sheep in Patagonian rangelands have demonstrated that multi-sensor tags combining accelerometry, magnetometry, GPS, and GIS landscape layers can quantify detailed behavioral patterns, feeding rates, and energy costs under rangeland conditions, although such systems remain expensive and energy-demanding (23). More broadly, a comprehensive review of precision livestock management on rangelands has outlined how real-time GPS tracking and accelerometer technologies can support grazing distribution management, disease detection, and parturition monitoring in extensive beef and sheep operations, while noting that cost and connectivity remain key barriers to widespread adoption (21). These considerations motivate the present review’s focus on extensive systems and inform the critical evaluation of evidence throughout the following sections.

## 2 Modalities in animal health monitoring

**Figure 1:**
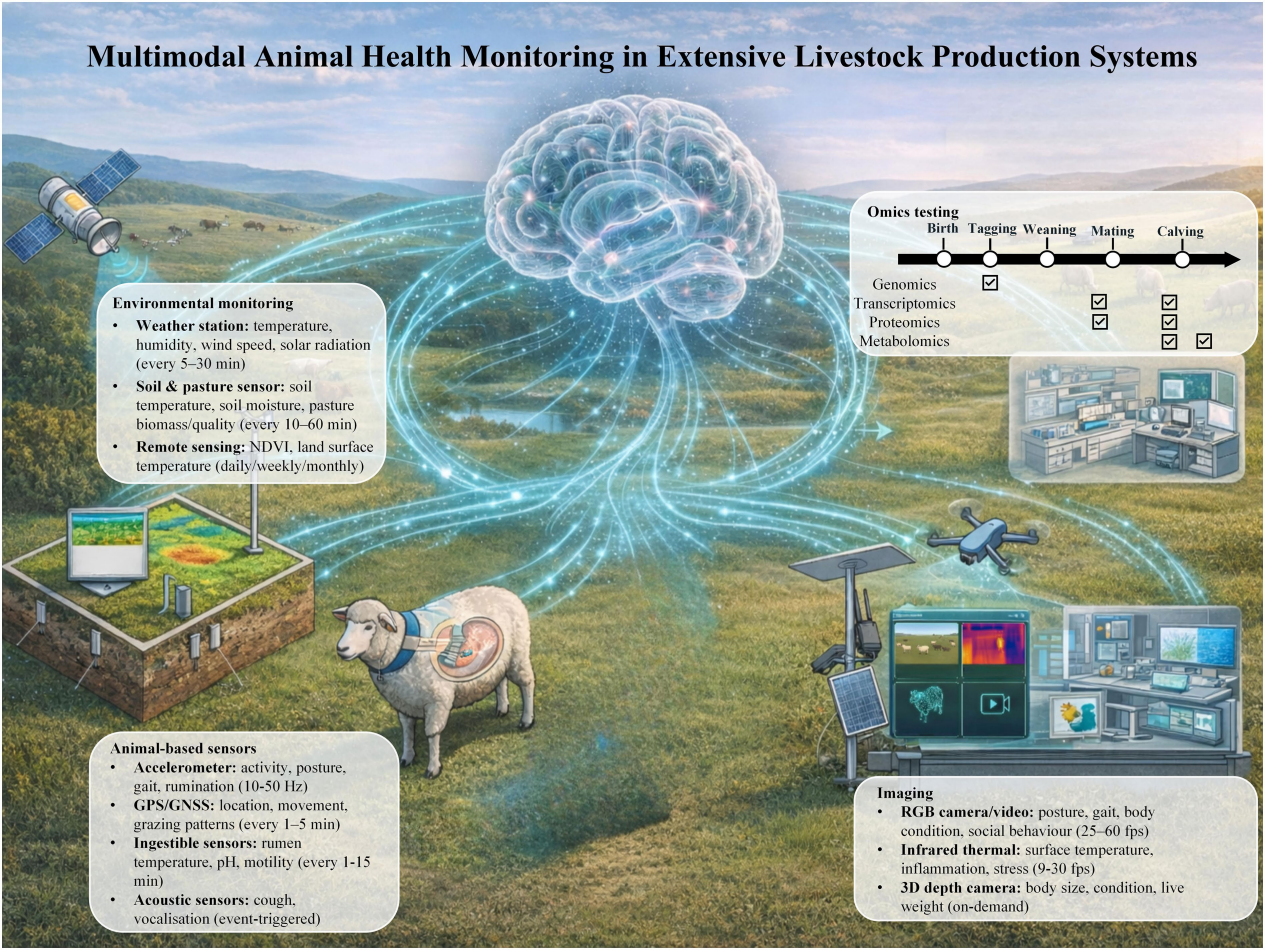
Multimodal animal health monitoring in extensive livestock systems. Schematic overview of integrated data streams, including imaging, wearable and ingestible sensors, omics technologies, and environmental monitoring. These inputs are combined within an artificial intelligence and machine learning framework to support early disease detection, welfare assessment, performance prediction, and decision support in extensive livestock systems.

Multimodal monitoring integrates complementary data streams that capture diverse and interconnected aspects of animal behavior, health, performance, and environmental exposure (Figure 1). Each modality provides a distinct layer of information, ranging from continuous behavioral and physiological measurements to high-resolution molecular and environmental signals. When combined, these modalities enable a more comprehensive and dynamic understanding of animal health and disease processes than can be achieved by any single data source alone.

### 2.1 Environmental monitoring

Environmental monitoring provides a critical contextual layer for interpreting animal health and performance in extensive livestock systems. Rather than serving as background information, environmental data can quantify the external pressures, such as climate variability (24), resource availability (25), and landscape heterogeneity (26), that directly influence disease risk, physiological stress, and overall animal resilience (3). Continuous or high-frequency measurements from on-animal, in-paddock, and remote sensing platforms enable detailed characterization of the conditions experienced by animals in real time, which is essential in systems where direct observation and handling are limited.

Proximal sensing technologies, such as stationary weather stations and on-animal loggers, capture micro-climatic variables, including ambient temperature, humidity, wind speed, and solar radiation. While some in-animal devices also record behavioral data, their environmental sensing capability provides essential context for interpreting animal responses. These factors directly influence thermoregulation (27), feed and water intake (28–30), and behavior (31). They are also key drivers of heat stress (32, 33) and reduced productivity (34, 35). In extensive systems, fine-scale environmental variation, arising from differences in terrain, resources, and access to shade and shelter, can result in substantial heterogeneity in exposure within a single herd (36). Such variation is often overlooked in traditional monitoring but can lead to differential disease susceptibility and health and welfare outcomes among animals (37).

Recent advances in AI-driven Earth observation, such as AlphaEarth (38) and TESSERA (39), have produced foundation models that generate analysis-ready environmental representations at high spatial resolution. At broader spatial scales, remote sensing provides scalable assessment of environmental conditions across large grazing areas. Satellite-derived indices such as the Normalized Difference Vegetation Index (NDVI) provide proxies for pasture biomass and quality (40), which are closely linked to nutritional status and susceptibility to metabolic disorders. However, the temporal resolution of satellite revisit cycles may limit the detection of rapid pasture changes, such as those driven by short-duration grazing events or sudden weather shifts. In addition, validating pasture growth and biomass estimation remains particularly challenging in botanically diverse swards on hill country terrain, given that most existing research has been conducted on ryegrass-dominant pastures on flat or gently undulating land. In parallel, land surface temperature (LST) and soil moisture offer indicators of thermal load (41) and feed availability (42, 43), both of which influence stress responses and disease risk. These data enable monitoring of spatial and temporal variation in environmental stressors, including drought, heatwaves, and pasture decline, which are major drivers of health challenges in extensive livestock systems.

Environmental monitoring becomes most valuable when integrated with animal-based sensor and molecular data. The power of this integration lies in modeling interactions, linking environmental conditions with behavioral, physiological, and molecular responses to explain variation in health outcomes. This enables key questions to be addressed. For example, such integration can help determine whether genomic markers for heat tolerance confer advantages only under specific environmental thresholds (e.g., periods of high thermal load or low pasture availability), or whether transcriptomic signatures of immune activation following environmental stressors can predict subsequent clinical disease. By connecting molecular and phenotypic responses to precise environmental conditions, multimodal systems move beyond describing observed changes to identifying their underlying drivers and predicting which animals are most at risk under dynamic and often challenging conditions. For producers, this translates into more timely and informed management decisions, enhancing production efficiency and animal welfare.

### 2.2 Animal-based sensors

Animal-based sensing technologies provide continuous, high-frequency measurements of behavior and physiology, offering insights into animal health that are not readily captured through visual observation alone. Deployed directly on or within the animal, these systems primarily include wearable and ingestible devices, enabling real-time monitoring of both external behavioral patterns and internal physiological states.

Wearable devices are widely adopted in livestock systems and typically incorporate motion and location sensors, such as accelerometers and gyroscopes, embedded in collars, ear tags, or leg-mounted devices. These sensors enable continuous quantification of activity and posture, facilitating the classification of behaviors including grazing, rumination, lying, standing, and locomotion. By establishing individual behavioral baselines, wearable systems can detect subtle deviations associated with disease onset. For example, reduced activity levels, altered gait dynamics, and changes in lying behavior have been linked to lameness (44). Similarly, disruptions in feeding and rumination patterns are associated with metabolic disorders (45) and mastitis (46). Empirical studies have demonstrated that wearable sensors can detect a range of health conditions, including lameness (44), metritis and clinical mastitis in cattle (47), neonatal calf diarrhea (48), and parasitism in lambs (49).

Many wearable platforms also incorporate Global Navigation Satellite System (GNSS) receivers, enabling spatial tracking of animal movement and grazing behavior. These data provide valuable insight into feed utilization (50), movement patterns (51), and social interactions (52). Deviations from typical movement behavior, such as reduced mobility or separation from the herd, may serve as early indicators of compromised health or welfare.

Wearable sensing technologies are increasingly transitioning from research to commercial deployment. Several platforms incorporating accelerometer-based monitoring are currently available for cattle (e.g., Halter, CowManager, eShepherd, Nedap), providing continuous behavioral tracking and automated health alerts. Some have demonstrated the ability to detect behavioral changes several days prior to observable clinical signs (53). Despite these advances, commercially validated solutions remain largely limited to cattle, with few options available for other livestock such as sheep. This represents a key gap, especially for extensive livestock systems where labor constraints make automated monitoring essential. For instance, a review of precision livestock farming applications in extensive dairy sheep farming concluded that while technologies such as accelerometer-based pedometers and GPS/GIS tracking offer considerable promise for flock management in pastoral systems, their operational deployment remains limited by high per-unit sensor cost, energy supply requirements, and the lack of decision-support tools tailored to production systems (53).

Ingestible sensors, commonly deployed as rumen boluses or capsules, provide direct measurements of internal physiological parameters, including core rumen temperature, rumen pH, and reticulorumen motility (2, 54, 55). These devices enable continuous monitoring of thermoregulation and digestive function, which are critical components of animal health. For instance, elevated internal temperature can indicate infection or systemic inflammation, while changes in rumen pH and motility are associated with digestive disturbances such as subacute ruminal acidosis. In addition, patterns of rumen contractions and temperature fluctuations can be used to infer rumination and drinking behavior, offering further insight into metabolic status.

Although not strictly animal-based, acoustic sensing provides an additional, complementary modality for monitoring physiological states that are not directly captured through movement or internal measurements. Microphone-based systems can detect and classify sound patterns associated with respiratory disease, particularly through automated recognition of coughing events (56, 57). These approaches have demonstrated the potential to identify respiratory infections, such as bovine respiratory disease, several days prior to the onset of observable clinical signs. Analysis of vocalizations has also been explored as an indicator of animal welfare, with machine learning models used to characterize vocal patterns associated with stress, discomfort, or social isolation (58). Although acoustic systems are commonly deployed at the group or environment level rather than on individual animals, they provide valuable population-level signals that complement animal-based sensing.

Together, wearable, ingestible, and acoustic sensing modalities provide a continuous and multidimensional view of animal behavior and physiology. However, each modality captures only a subset of the underlying biological processes. Integrating these data streams is therefore essential to achieve the sensitivity and specificity required for early disease detection and informed decision-making in livestock management.

### 2.3 Imaging

Imaging technologies play a central role in non-invasive animal health monitoring by capturing visual, thermal, and structural information at different spatial resolutions. Standard digital cameras that capture red, green, and blue (RGB) wavelengths are the most widely used and cost-effective imaging tools. Note that video is a temporal extension of static imaging rather than a separate modality. Dynamic behaviors such as feeding, locomotion, and social interactions require continuous frame sequences to capture temporal patterns (59). These systems form the foundation for analyzing macroscopic characteristics such as posture, gait, and body condition. In livestock systems, RGB camera-based technologies are already commercially available to assess locomotion and detect lameness in cattle (60). However, conventional RGB imaging lacks depth information, limiting its ability to accurately quantify three-dimensional structures (61, 62). In addition to this inherent technical limitation, deploying imaging systems within extensive livestock systems introduces a distinct set of operational challenges that fundamentally restrict their practical utility. Unlike controlled indoor facilities, open pastures are characterized by highly variable illumination, complex and dynamic backgrounds, inter-animal occlusion within mobile flocks, and motion blur. These factors substantially degrade the performance of image-based algorithms predominantly developed and validated in indoor barn environments (63).

To address the lack of depth information in conventional RGB imaging, three-dimensional (3D) imaging technologies, including depth cameras and structured light systems, are increasingly used to capture volumetric information (62, 64). These approaches enable more accurate estimation of body size, body condition, and live weight, key indicators of animal health and welfare, in both cattle (65) and sheep (66). By reconstructing 3D morphology, these systems provide a more objective and repeatable alternative to visual scoring.

Infrared thermography (IRT) offers a complementary modality by enabling non-contact measurement of surface temperature. In veterinary applications, IRT cameras convert emitted radiation from the animal’s skin surface into thermographic images (67). This technique has demonstrated utility in detecting inflammatory and infectious conditions, including mastitis in cattle (68) and sheep (69), hoof health in both species (70, 71), bovine viral diarrhea (72), bovine respiratory disease (73), bluetongue virus in sheep (74), and neonatal calf diarrhea (75). Beyond disease detection, IRT is also used as a non-invasive indicator of animal welfare (76). For example, changes in eye temperature have been associated with autonomic nervous system activation in response to stress and pain in both sheep (77) and cattle (78). Respiration rate and heat stress can also be inferred from thermal signals (79) enabling early detection of thermal load before clinical signs emerge and supporting timely cooling or shade interventions that protect both welfare and productivity.

Despite its advantages, IRT measures only surface temperature and is sensitive to environmental and physiological confounders, including ambient temperature, airflow, coat characteristics, and metabolic state. As such, standardization of measurement conditions and careful interpretation are essential for reliable application. Nevertheless, IRT remains a rapid, low-cost, and animal-friendly tool that can be integrated into routine veterinary assessments with minimal handling (80).

Ultrasound imaging provides an additional and clinically established modality for assessing internal tissue structure and function (81). Widely used in veterinary practice, ultrasound enables real-time evaluation of reproductive status, such as pregnancy diagnosis (82), muscle and fat depth (83, 84), and internal organ health (85–87). In livestock production, it is commonly applied to estimate carcass traits such as backfat thickness and muscle area, supporting both health assessment and breeding decisions (88, 89). While ultrasound offers deeper anatomical insight than surface imaging techniques, its application in extensive systems is often constrained by the need for close animal contact, operator expertise, and handling infrastructure. However, emerging portable and automated ultrasound systems present opportunities for integration into broader multimodal monitoring frameworks (88).

In addition to visible and thermal imaging, multispectral and hyperspectral imaging extend sensing capabilities across a wider range of the electromagnetic spectrum, including near-ultraviolet, visible, and infrared bands. These technologies capture detailed spectral signatures at the pixel level, enabling detection of subtle physiological and biochemical changes. In livestock systems, hyperspectral imaging has shown promise in abattoir settings for automated detection of defects in carcasses and offal, offering a non-invasive and efficient alternative to manual inspection (90).

Collectively, these imaging modalities have distinct strengths and limitations, exhibiting complementary failure modes. For example, RGB imaging provides high-resolution texture but performs poorly under low-light conditions, whereas thermal imaging is robust to lighting but lacks fine spatial detail. Neither modality alone can accurately capture volumetric structure, which is addressed by 3D imaging. Ultrasound, in turn, provides internal anatomical information but is less scalable in extensive systems. These complementary characteristics highlight the need for multimodal integration, where combining imaging modalities can provide a more robust, comprehensive, and scalable approach to animal health and welfare monitoring.

### 2.4 Omics

Omics data provide a critical molecular layer that complements the environmental, sensor-based, and imaging modalities described above. While other modalities capture exposure, behavior, and phenotype, omics approaches uncover the underlying biological mechanisms that drive these changes. This enables a shift from descriptive monitoring toward mechanistic understanding and early prediction of disease processes in extensive livestock systems.

A key strength of omics data lies in their ability to capture both inherent biological potential and dynamic responses to environmental and physiological stressors. Genomic information provides stable, lifelong insight into an animal’s susceptibility or resistance to disease. Key examples include traits such as parasite resistance in sheep (91, 92), mastitis resistance in both dairy cattle (93) and sheep (94), and tolerance to heat stress (95). These genomic signals underpin breeding strategies aimed at improving herd-level resilience and long-term animal health outcomes.

In contrast, transcriptomic, proteomic, and metabolomic profiles reflect real-time biological activity, offering sensitive indicators of early or subclinical disease (15). For example, transcriptomic responses associated with immune activation can precede visible signs of infection, enabling earlier detection of conditions such as bovine respiratory disease (96) or gastrointestinal parasitism (97, 98). Proteomic biomarkers, including acute-phase proteins and inflammatory mediators, have been used to detect mastitis (99, 100), metabolic disorders (101, 102), and systemic inflammation (100, 103) before clinical diagnosis. Similarly, metabolomic profiles provide insight into energy balance and metabolic function, supporting early identification of conditions such as negative energy balance in dairy cattle (104, 105) or subacute ruminal acidosis (106, 107) in grazing systems.

The microbiome adds an additional layer of biological context, particularly in ruminant livestock where microbial communities play a central role in digestion, nutrient utilization (108), as well as immune function (109). Changes in rumen microbial composition have been linked to feed efficiency (110, 111), methane emissions (112, 113), and susceptibility to metabolic disorders (114–116). For instance, shifts in fiber-degrading microbial populations can signal disruptions in rumen function, while alterations in microbial diversity may reflect dietary transitions or disease challenges (117, 118). These microbial signals are particularly powerful when interpreted alongside behavioral data (e.g., feeding patterns) and environmental conditions (e.g., pasture quality and climate variability).

Importantly, the value of omics data is maximized when interpreted in conjunction with other modalities. For example, integrating rumen microbiome profiles with feeding behavior derived from wearable sensors and pasture quality estimated from remote sensing can provide early warning of digestive inefficiencies or impending metabolic disorders. Similarly, combining genomic risk profiles with continuous body temperature and activity data from sensors can help identify animals that are more susceptible to heat stress or infectious disease under specific environmental conditions. Likewise, proteomic biomarkers of inflammation, when interpreted alongside thermal imaging data, could improve the specificity of early mastitis detection by distinguishing true inflammatory responses from environmental heat effects.

Despite their potential, the application of omics technologies in extensive livestock systems remains constrained by cost, sampling logistics, and challenges in data integration and interpretation. However, advances in high-throughput technologies, decreasing sequencing costs, and the development of portable and field-deployable assays are increasing the feasibility of incorporating omics into multimodal monitoring frameworks.

### 2.5 Multimodal animal health monitoring

As outlined above, each modality captures only a partial view of an animal’s health status. Wearable sensors quantify behavioral deviations but cannot identify their underlying cause; thermal imaging detects surface temperature anomalies yet is confounded by environmental conditions; and omics profiling reveals molecular mechanisms but is typically available at lower sampling frequency due to practical sampling constraints. As an illustrative example, elevated surface temperature from infrared thermography, together with reduced activity from accelerometer data and inflammatory biomarkers from proteomic profiling, could provide more convincing evidence of an emerging infection, with the real-time sensor signals enabling immediate on-farm response and the molecular layer subsequently confirming etiology. This complementarity is likely important for improving diagnostic sensitivity in extensive systems, where limited direct observation increases reliance on automated integration of diverse data streams (6, 21, 119). The following section examines the technical strategies by which these heterogeneous modalities can be aligned, fused, and jointly modeled to realize this potential.

## 3 Data integration strategies

**Figure 2:**
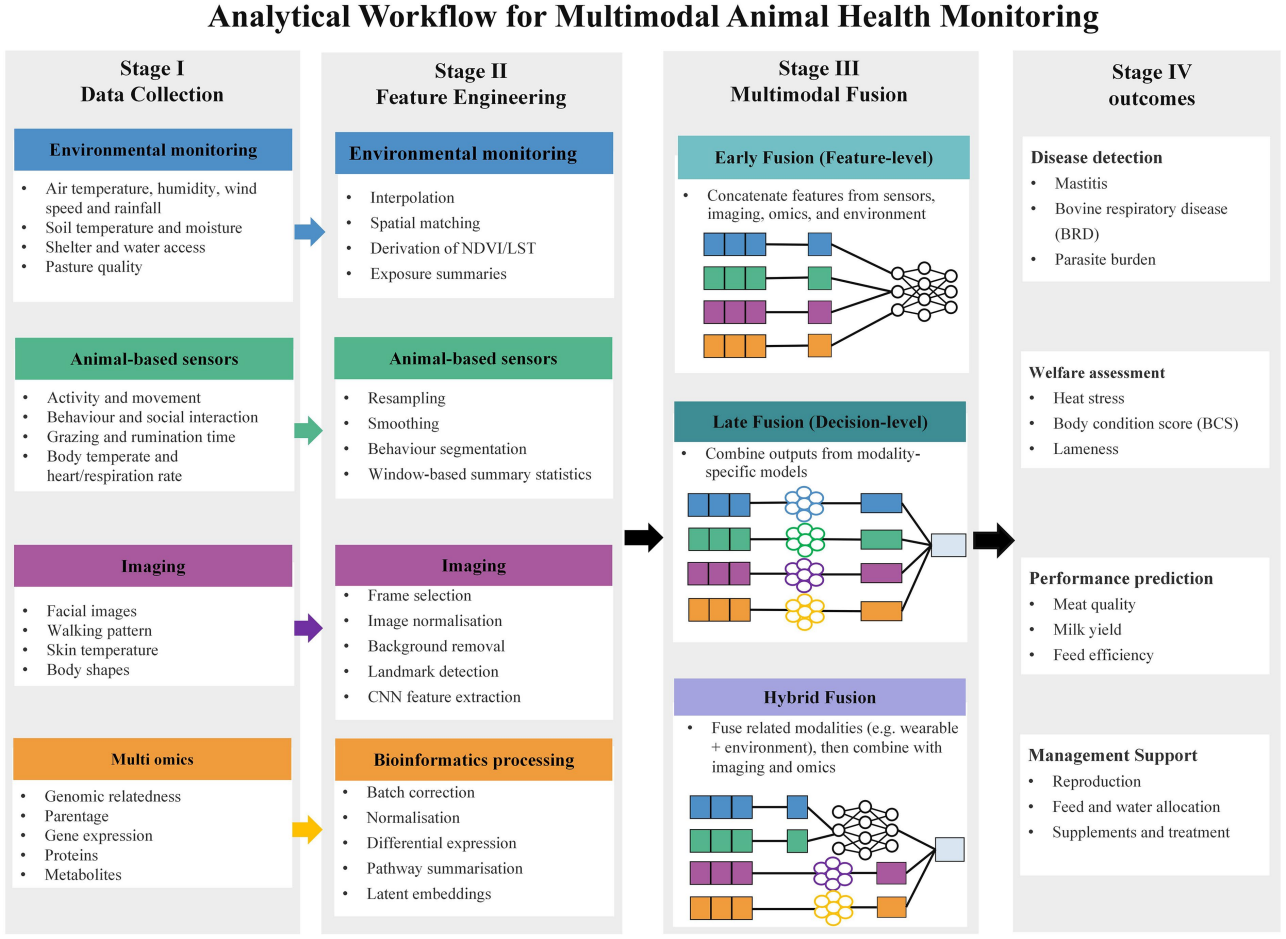
Multimodal data processing pipeline for animal health monitoring in extensive livestock systems. Schematic representation of a four-stage pipeline integrating heterogeneous data streams for animal health assessment. Stage 1 (Data collection) includes imaging (e.g., RGB, thermal, 3D), sensor-derived behavioral and physiological data, omics profiles, and environmental measurements. Stage 2 (Feature processing and selection) involves data quality control, cleaning, and normalization, followed by modality-specific feature extraction, including convolutional neural network (CNN)-based image features, spatial–temporal sensor signals, molecular biomarkers from omics data, and contextual environmental variables. Stage 3 (Multimodal fusion) combines these features using early (feature-level), late (decision-level), or hybrid fusion strategies to integrate complementary information across modalities. Stage 4 (Prediction and outcome) applies predictive models to support disease detection, welfare assessment, performance prediction, and early warning. This framework highlights the role of multimodal integration in enabling more robust and comprehensive livestock health monitoring.

The efficacy of any multimodal animal health monitoring system is fundamentally constrained by the quality and interoperability of its underlying data. Feature engineering extracts meaningful patterns from high-frequency raw sensor data to characterize specific biological states, while data integration combines these patterns with environmental and health records. In doing so, these techniques transform raw, heterogeneous data streams, from wearable sensors, imaging systems, omics, and environmental monitors, into a cohesive, informative representation for predictive modeling. This process faces several technical challenges, including disparate sampling rates, inconsistent data quality, and missing values due to device failure or censoring. Additionally, high-dimensional feature spaces can cause some modalities to dominate others (120). Effective integration strategies are therefore not merely a final analytical step but a critical preprocessing and alignment pipeline (Figure 2) that determines the ceiling of system performance. This section reviews reported methods for data alignment and processing, details the primary paradigms for fusing information, and explores advanced concepts like transfer learning and agentic AI that may enhance robustness and generalizability across the unpredictable conditions of extensive livestock systems, although empirical evidence for their effectiveness in this domain remains limited.

### 3.1 Preprocessing

Before fusion can occur, raw data streams from different modalities require meticulous synchronization, cleaning, and transformation into a compatible format. A primary challenge in extensive systems is temporal alignment, as data are collected at vastly different frequencies. For example, GPS positions may be recorded at sub-second intervals, but environmental data are collected daily. A useful strategy is time-windowing, where all signals within a defined period are aggregated into summary statistics (mean, variance, frequency-domain features) (121).

A more sophisticated challenge is handling missing data and unaligned modalities, which is pervasive due to device loss, battery failure, or the sheer cost of collecting certain data types. Traditional imputation methods (mean, median, *k*-nearest neighbors) may be inadequate for complex, high-dimensional data (122, 123). Recent advances in generative models offer a powerful alternative. Frameworks such as Generative Adversarial Imputation Nets (124) use an adversarial learning scheme to create a joint embedding space from unaligned data with missing modalities. Its adversarial architecture can generate a plausible representation of a missing modality from an available one, maintaining informational consistency. This approach is directly transferable to livestock contexts, for example, generating synthetic behavioral feature sets from partial genomic profiles to compensate for temporary sensor failure (18).

### 3.2 Fusion architectures

The architecture of integration dictates how relationships between modalities are learned. These are generally categorized into three paradigms: early (feature-level), late (decision-level), and hybrid fusion.

#### 3.2.1 Feature-level fusion

Feature-level fusion involves the direct concatenation of raw or engineered features from different modalities into a single, unified feature vector, which is then fed into a machine learning model (119). This approach allows the model to learn complex, non-linear interactions between modalities directly from the data, potentially uncovering novel composite features. For instance, a model might learn that a specific combination of a slight increase in nocturnal activity and a subtle change in vocalization frequency is a more reliable early indicator of respiratory distress than either signal alone. However, feature-level fusion is computationally intensive and sensitive to data quality and scale. A significant risk is that models may become biased toward modalities with inherently higher dimensionality or less noise (120). Therefore, robust feature-level integration depends critically on the rigorous alignment and processing pipelines described in Section 3.1.

#### 3.2.2 Decision-level fusion

Decision-level fusion offers a more flexible and often more robust alternative by combining the final outputs (predictions or decisions) of separate unimodal models. In this paradigm, individual models, developed within one data modality, function as a committee that votes on the final health classification (125). Common ensemble techniques include weighted voting, averaging, or stacking with a meta-learner. This approach is particularly advantageous when data quality varies, as is common in extensive livestock systems. If one sensor fails, its corresponding model may be deprioritized or omitted without necessitating a complete system retraining, allowing others to provide a baseline prediction (126). The trade-off for this robustness and flexibility is a reduced capacity to discover novel cross-modal interactions, as the models operate in isolation until the final decision stage. Nevertheless, for many practical applications with high risks of data loss, decision-level fusion provides a reliable and implementable integration strategy.

#### 3.2.3 Hybrid approaches

Hybrid approaches combine elements of feature- and decision-level fusion (127, 128). For instance, wearable and environmental features may be fused at the feature level (121, 129), while omics and imaging models contribute separately at the decision level (130, 131). This layered integration can provide flexibility, balancing the richness of interaction modeling with robustness to missing modalities.

### 3.3 Representation learning and embeddings

Deep learning and representation learning methods enable the extraction of low-dimensional embeddings from high-volume data sources such as omics profiles or video streams (132, 133). These embeddings can then be integrated with features from other modalities, reducing dimensionality while preserving key information. For example, convolutional neural networks can generate embeddings of locomotion patterns from drone footage, which can be combined with rumination features from sensors and genomic resilience scores (134). Such methods are promising but require large, well-curated datasets that are not yet widely available in extensive livestock contexts.

### 3.4 Model training strategies

Training multimodal models for extensive livestock systems presents unique challenges compared to standard computer vision or sensor tasks, primarily due to data scarcity and class imbalance.

#### 3.4.1 Transfer and multi-task learning

Given the scarcity of labeled data in extensive livestock systems, transfer learning is essential (135). Labeled data refers to datasets in which each sample has been manually annotated with a known outcome, for example, an image of a cow tagged as “lame” or “healthy”, enabling supervised models to learn the mapping between inputs and target conditions. Models trained on dairy cows in confined systems can be fine-tuned to detect lameness in extensive beef cattle (136). Furthermore, multi-task learning frameworks can simultaneously predict multiple outcomes (e.g., heat stress and parasite burden) from a shared representation. This encourages the model to learn generalized features that are robust across different health challenges, maximizing the utility of limited datasets.

#### 3.4.2 Self-supervised learning (SSL)

To address the bottleneck of manual annotation, SSL is emerging as a critical training paradigm (137). By creating pretext tasks, such as masking sections of sensor data and training the model to reconstruct them, algorithms can learn robust feature representations from vast amounts of unlabeled data (138). These pre-trained encoders can then be fine-tuned on small, labeled datasets for specific disease detection tasks.

### 3.5 Benchmarking and evaluation

Establishing robust evaluation frameworks is essential for comparing multimodal monitoring systems and assessing their readiness for field deployment. However, evaluation practice in the literature remains inconsistent in both the metrics selected and the validation protocols employed. Table 1 summarizes evaluation metrics commonly used in multimodal livestock health monitoring models, organized by task type. Understanding the strengths and limitations of each metric is critical for designing appropriate validation protocols and interpreting reported results.

**Table 1:** Summary of evaluation metrics commonly used in multimodal livestock health monitoring models.

Metric	Type	Description
Accuracy	Classification	Measures the proportion of total predictions that were correct.
Precision	Classification	Measures the proportion of positive predictions that were actually correct.
Recall (sensitivity)	Classification	Measures the proportion of actual positive cases that were correctly identified.
*F*1-score	Classification	Provides a single balanced score by calculating the harmonic mean of precision and recall.
AUC-ROC	Classification	Represents the model’s ability to distinguish between positive and negative classes across all thresholds.
Specificity	Classification	Measures the proportion of actual negative cases (healthy animals) that were correctly identified.
Root mean square error (RMSE)	Regression	Calculates the square root of the average of squared differences between predicted and actual values.
*R*-squared (*R*^2^)	Regression	Indicates the percentage of the variance in the dependent variable that the independent variables explain collectively.
Mean absolute error (MAE)	Regression	Calculates the average of the absolute differences between predicted and actual values.
Mean average precision (mAP)	Detection	Computes the average of the average precision (AP) scores across all classes for a detection task.
Detection latency	Early warning	Quantifies the advance warning time provided between a system alert and the onset of clinical signs.

Metrics are grouped by task type, including classification, regression, object detection, and early warning, with descriptions of their role in assessing model performance for extensive livestock systems.

Beyond metric selection, the choice of validation protocol is equally consequential. Naive random splitting of data (e.g., 80/20 train/test) is often invalid in livestock monitoring due to high temporal correlation and individual variance. To ensure generalizability, evaluation should employ leave-one-subject-out or leave-one-group-out cross-validation (121). This prevents the model from “memorizing” the identity or specific movement anomaly of individual animals, ensuring that the reported performance reflects the system’s expected performance if new, unseen animals are enrolled for diagnosis (22). Beyond validation protocol design, the gap between controlled-environment performance and real-world deployment remains poorly documented. A systematic review of commercially available precision livestock farming technologies for dairy cattle welfare found that only 5% had undergone external validation on populations different from those used during system development (22). This finding highlights a pervasive issue: most reported performance metrics reflect in-sample validation and may substantially overestimate accuracy. Under field conditions, sensor fouling, device loss, and environmental variability degrade data quality over time. Table 2 provides a chronological overview of representative multimodal studies, detailing the species investigated, data modalities combined, metrics reported, and validation protocols employed.

**Table 2:** Summary of representative multimodal studies in extensive livestock systems.

No.	Year	Species	Modality	Evaluation metrics	Validation	Reference
1	2023	Cattle	Accelerometer; GPS; environmental data	Accuracy 88.5%; *F*1	Leave-one-animal-out (LOAO) Cross-validation (CV)	(171)
2	2023	Cattle	Accelerometer; GPS	*R*^2^ = 0.99; RMSE = 1.6 min; drinking *R*^2^ = 0.90; concentrate feed *R*^2^ = 0.85	Comparison with accelerometer-only baseline	(172)
3	2023	Pig	Acoustic; IRT	Accuracy 99.77%	5-fold CV	(173)
4	2024	Cattle	Accelerometer; RGB; environmental data	mAP (behavior detection); RMSE (milk yield); accuracy (behavior recognition);	Multi-baseline benchmark evaluation	(174)
5	2024	Cattle	Accelerometer; video	Accuracy 98.80%; precision 97.15%; recall 96.93%	K-fold CV	(166)
6	2024	Pig	Acoustic; IRT	Accuracy 98.79%	Feature selection via cross-validated RFE; evaluation on test data	(175)
7	2024	Sturgeon	RGB; IRT; accelerometer	Accuracy 88.96%; precision 90.06%; recall 89.43%; *F*1 89.49%	K-fold CV	(176)
8	2025	Cattle	Accelerometer; environmental data	Welfare scoring accuracy; expert agreement; BP neural network validation	Comparison with traditional single-modality welfare scoring	(167))

For each study, the table lists the target species, modalities, evaluation metrics reported, and validation approach employed. Studies are ordered chronologically, illustrating the breadth of multimodal applications across animal production systems.

### 3.6 Agentic AI and future opportunities

A transformative frontier in integration is the move from static fusion architectures to adaptive, agentic AI systems. These systems can reason about context, manage uncertainty, and dynamically alter their integration strategy in response to real-time conditions and goals (128).

In animal health monitoring, an agentic AI could potentially perform context-aware modality prioritization. For example, during a severe dust storm, visual drone data becomes unreliable. The AI agent could potentially dynamically down-weight predictions from the vision model and increase reliance on wearable physiological sensors and stationary environmental stations. Frameworks like Mastra illustrate the technical architecture for such agents, featuring a multimodal perception hub for synchronized data parsing and a dynamic knowledge engine that updates decision logic based on new information. An agentic AI for livestock could, in theory, integrate pasture quality forecasts, market prices, and individual animal health predictions to make prescriptive management recommendations. Looking forward, rumen boluses or ear-tag sensors could measure body temperature per animal, with concurrent elevations across multiple animals signaling a potential herd-level event rather than a single outlier. The agent could initiate an action, such as tasking a drone to perform a targeted thermal imaging flight to identify the most severely affected individuals. Such autonomous decision-making capabilities have not yet been built in extensive livestock production systems, and substantial challenges remain in ensuring reliability, interpretability, and safety of agent-driven actions at commercial scale.

Core technical challenges, such as sensor fusion, missing data, and individualized prediction, are shared across animal and human health domains. Recent advances in multimodal learning, including adversarial training frameworks such as MIRAGE (139) and dynamic fusion architectures such as REI-net (140), have been largely driven by bioinformatics and computational vision research focused on human health. Similarly, the emergence of agentic AI for personalized health management supports advances in continuous human health monitoring and “hospital-at-home” models (141). These advances provide a conceptual foundation for developing analogous systems in livestock, though direct translation to extensive systems will require addressing domain-specific challenges including data scarcity, environmental variability, the absence of standardized livestock health benchmarks, and data governance constraints that limit data sharing across farms, commercial providers, and research institutions.

## 4 Practicality and deployment

The technical elegance of multimodal integration strategies forms the computational foundation for advanced health monitoring, yet their ultimate success in extensive livestock systems hinges on a factor of equal importance: practicality. A system that achieves high predictive accuracy in controlled research trials will fail if it is not affordable, durable, and seamlessly usable by farmers and veterinarians. In these environments, practicality is as important as technical performance, determining whether a technology remains a prototype or becomes a trusted tool for daily management.

### 4.1 Cost and scalability

Extensive systems involve flocks or herds distributed across wide areas. While equipping every animal with advanced sensors or performing routine omics sampling could inform target management decisions, such approaches are often cost-prohibitive in extensive systems (142). Deployment strategies must therefore consider scalability, striking a balance between per-animal resolution and overall herd coverage. Hybrid approaches, such as equipping sentinel animals with wearable devices or focusing molecular sampling on high-risk cohorts, may improve feasibility without compromising insight. However, even targeted monitoring strategies generate substantial data volumes, imposing significant hardware constraints (143). The transition to continuous animal tracking requires high-performance computing (HPC) clusters, specialized graphics processing units (GPUs), and vast storage capacities to manage the results (144). For many operations, the high capital expenditure required for this advanced computer hardware and the energy costs associated with maintaining such infrastructure represent a critical disadvantage that can hinder the practical adoption of precision livestock farming technologies (145). Limited evidence on economic returns can further complicate investment decisions. An analysis of farm accounting data from 217 Dutch dairy farms found that sensor investment did not translate into measurable improvements in farm profitability, implying that economic benefits depend more on how sensor information is being utilized in management (146, 147). A conceptual framework for assessing the value of information from precision livestock farming has highlighted that profitability is largely driven by detection accuracy, herd size, and the farmer’s capacity to act on alerts (148). A study eliciting dairy farmers’ willingness to pay for monitoring devices found that adoption is more likely when farmers perceive devices as easy-to-use, although cost remains a primary constraint for small and medium enterprises (146).

### 4.2 Connectivity and infrastructure

Extensive systems often lack reliable network coverage, limiting the feasibility of real-time data transfer. This constraint motivates alternative solutions such as edge computing (149), where preliminary processing occurs on the device or at the paddock level, and only summary outputs are transmitted when connectivity is available. Systems must therefore be designed to tolerate intermittent data flows and degrade gracefully rather than fail outright when connectivity is lost. For active wearables that influence animal movement, such as virtual fencing collars, fail-safe design is equally important: devices should default to a state that preserves animal welfare when communication is disrupted, ensuring that animals retain access to water, shade, and shelter.

Beyond connectivity, long-term sensor reliability presents a further challenge. Concept drift, where changes in animal behavior, seasonal conditions, or sensor aging cause the underlying data distribution to shift, can substantially undermine the performance of models trained under existing conditions, requiring systematic quality-control procedures (150).

Despite their practical importance, sensor failure rates, maintenance burden, and recalibration requirements remain underreported, constraining the assessment of operational cost and reliability in multimodal animal health monitoring systems deployed under field conditions.

### 4.3 Labor and management burden

A core promise of digital technologies is to reduce, rather than increase, on-farm workload; however, complex device installation, frequent maintenance, and high data-management demands can deter adoption. Systems should therefore minimize labor requirements, be simple to deploy across large herds/flocks, and integrate seamlessly into existing farm management workflows. Automated alerting and prioritization of at-risk animals can help ensure that technology supports, rather than competes with, day-to-day decision-making.

Qualitative research supports this principle, with semi-structured interviews in the swine industry indicating that technology perceived to increase the workload is unlikely to be adopted regardless of its technical merits (151). Similarly, applications of Normalization Process Theory in sheep farming show that adoption depends not only on perceived usefulness but also on how readily tools can be embedded within existing management routines (152). These findings underscore the importance of designing animal health monitoring systems that complement, rather than disrupt, established practices.

### 4.4 Usability and interpretability

For farmers and veterinarians, actionable insights are more valuable than complex predictive scores. Outputs must be presented in formats that are intuitive and directly linked to management actions, such as traffic light indicators, risk alerts, or ranked animal lists (153). This shift toward interpretability not only facilitates decision-making but also builds essential trust in the technology (154). Farmers are more likely to adopt and rely on systems whose recommendations can be understood and justified within their specific operational context. Conversely, low-specificity alerts risk generating alert fatigue and reducing farmer engagement with the system. Effective design must therefore prioritize meaningful signal extraction over raw data volume (155).

### 4.5 Integration with existing systems

Monitoring technologies do not operate in isolation but must integrate with farm management software, animal identification systems, and veterinary reporting tools. Seamless interoperability reduces duplication of effort and ensures that health insights are embedded within broader decision-support platforms; standardization of data formats and communication protocols represents a key step toward integrated farm-wide ecosystems. Beyond technical interoperability, the governance of data generated by multimodal monitoring systems raises important questions about ownership, privacy, and equity. As farm data grow in volume and scope, control over these data confers increasing influence over management decisions, with evidence of consolidation among a small number of technology providers (156, 157). In extensive livestock systems, where producers often have limited bargaining power, there is a risk that data-driven tools may shift decision-making authority away from farmers. Clear frameworks for data custodianship, ownership, and governance are therefore critical to ensure that multimodal systems support, rather than undermine, producer autonomy.

### 4.6 Adoption barriers and enablers

As noted above, barriers to adoption include high upfront costs, uncertain return on investment (ROI) (147, 148), and limited access to training or technical support. Empirical studies consistently report these patterns across livestock sectors. For example, a survey of crop, dairy, and livestock producers in Wisconsin identified data privacy concerns, system incompatibility, and difficulty deriving actionable value from data as key adoption barriers, with adoption increasing alongside farm size, income, and younger producer age (158).

Data governance concerns further compound these challenges. Farmers report uncertainty around how data are collected, processed, and shared by technology providers, while the absence of clear legal frameworks for farm data protection exacerbates this reluctance (159). Without transparent data governance arrangements, there is a risk that the benefits of multimodal animal health monitoring will be disproportionately captured by technology providers rather than producers.

In ruminant systems, adoption is highest for technologies integrated into existing infrastructure (e.g., milking parlors), whereas stand-alone data-processing tools for disease detection remain underutilized, suggesting that ease of integration is a stronger driver than technical capability alone (160). Conversely, enablers of adoption include demonstrable improvements in welfare and productivity, co-design with farmers, and clear evidence from field validation. Adoption may also be driven by external market and compliance requirements, including assurance schemes, retailer expectations, welfare auditing, traceability, and environmental reporting, rather than solely by proactive farmer demand (156, 161).

Ultimately, practicality is not an inherent property of a technology, but an outcome of iterative, user-centered design, rigorous field validation, and the development of sustainable business models that align developer incentives with farmer success.

## 5 Discussion and future directions

Multimodal monitoring represents a significant advance in animal health management within extensive livestock systems. Unlike traditional approaches that rely on a single data stream, integration of signals from wearable sensors, imaging technologies, omics profiling, and environmental monitoring enables a multidimensional view of an animal’s health and welfare status. Sensors capture continuous behavioral and physiological patterns; imaging systems provide rich external phenotypes; omics reveals molecular processes underpinning resilience or vulnerability; and environmental data contextualizes these signals within climate, pasture, and landscape pressures. Table 3 summarizes the principal data modalities, their representative technologies and signal types, the analytical approaches commonly applied to each, and illustrative applications in livestock health and welfare assessment.

**Table 3:** Summary of data modalities and analytical approaches used in multimodal livestock health monitoring.

Modality	Data source/Technology	Signals captured	Analytical approaches	Application	References
Environmental	Weather, pasture sensors	Heat stress exposure	Time-series models	Heat stress prediction	(29, 33)
Wearable sensors	Accelerometer collars	Activity, posture	Classification models	Lameness detection	(51, 121)
Imaging	RGB/thermal camera	Body condition, inflammation	Computer vision	Welfare monitoring	(65, 70)
Omics	Proteomics, metabolomics	Immune response, metabolic state	Machine learning, biomarker models	Disease risk prediction	(15, 99)

Modalities include environmental monitoring technologies, physiological sensors, imaging systems, and molecular (omics). For each modality, the table lists typical data types, analytical methods, and representative applications in animal health and welfare assessment.

When combined, these modalities deliver a more complete and biologically grounded picture of health than any one source alone, offering unprecedented opportunities for earlier detection of disease, deeper understanding of resilience, and more proactive management of livestock in challenging environments. Many of these multimodal approaches draw on advances in human biomedical research (149). This cross-disciplinary exchange provides valuable methodological parallels while revealing critical gaps specific to livestock systems.

Despite this promise, several challenges remain. First, data heterogeneity complicates integration, as modalities differ in sampling frequency, dimensionality, and error structures; for example, accelerometer data may be collected at sub-second resolution, whereas omics sampling is typically sparse and event-driven. Second, data quality and incompleteness remain limiting, with sensors prone to signal loss, imaging sensitive to environmental conditions, and omics assays subject to technical variability. Third, robust benchmarking is still lacking, as many studies demonstrate proof-of-concept results without independent validation across diverse herds, environments, and production systems. Finally, translation gaps exist between academic breakthroughs and real-world deployment, particularly in extensive systems constrained by connectivity, labor, and infrastructure. These challenges are compounded by the absence of large-scale multimodal datasets and the sporadic nature of health events, which limits data capture at clinically relevant time-points.

A related challenge is the lack of systematic reporting on long-term system performance under field conditions. Most published studies evaluate models at a single time point, overlooking performance degradation due to physical wear, environmental exposure, and concept drift as animal and environmental conditions evolve (150). Without longitudinal benchmarks tracking prediction accuracy, sensor failure rates, and maintenance requirements over extended deployment, it remains difficult to assess whether multimodal animal health monitoring systems can sustain performance in practice. Establishing standardized reporting frameworks for deployment longevity and failure modes is therefore a key priority.

In addition, a representation gap exists between livestock health data and the large-scale datasets available in human domains, limiting the effectiveness of transfer learning and necessitating domain-specific modeling strategies. The economics of multimodal animal health monitoring adoption in extensive systems remain poorly characterized. Existing cost–benefit analyses are largely derived from intensive dairy operations, where higher per-animal value justifies investment (162). Whether comparable returns can be achieved in extensive beef or sheep systems, where margins are lower, remains an open question (146).

Looking ahead, several opportunities can advance the field:

1) Edge computing and the Internet of Things (IoT) are likely to play a central role in enabling multimodal monitoring in extensive systems. Devices deployed at the animal and/or paddock level with local processing capability can support near real-time anomaly detection in low-connectivity farms. By shifting computation from the cloud to the device, these systems reduce latency and extend battery life, making continuous monitoring viable in extensive grazing systems. Design considerations must account for the specific constraints of extensive systems. Successful deployment will require not only miniaturized and energy-efficient hardware but also algorithms robust to the greater environmental and behavioral variability inherent in rangeland production, where animals may range over large areas and direct observation is infrequent (21). In low-resource settings, where extensive systems predominate and infrastructure constraints are more pronounced, low-cost sensor packages combined with satellite-derived environmental data may offer a practical pathway for near-term implementation (23).
2) Federated learning offers a promising approach for enabling models to learn across multiple farms without centralizing raw data, thereby improving generalizability while preserving data privacy and ownership (163). However, practical implementation in extensive systems remains challenging due to heterogeneous data formats, non-independent and non-identically distributed data arising from differences in breed, management, and environment, as well as limited on-farm computational capacity and unreliable network connectivity in remote regions. These constraints suggest that performance gains observed in controlled or simulated settings may not directly translate to extensive livestock production systems.
3) Digital twins, virtual representations of individual animals that integrate omics, sensor, imaging, and environmental data, offer the potential to simulate health trajectories, predict risks, and support proactive management decisions (164, 165). However, current applications in livestock are limited to specific use cases, such as behavior recognition in housed dairy systems (166) and digital twin construction under incomplete modalities (167). The development of comprehensive, real-time digital twins for individual animals in extensive systems remains aspirational and will require advances in data infrastructure, model fidelity, and computational scalability.
4) Agentic AI represents a shift beyond passive data integration, with adaptive systems capable of managing monitoring workflows by prioritizing data streams, triggering additional measurements in response to anomalies, and suggesting interventions. Such approaches may help address the inherent unpredictability of extensive environments, acting as intermediaries between continuous digital data and intermittent human oversight (168).
5) Multi-objective optimization is needed. Future frameworks should not focus solely on accuracy, but explicitly balance sensitivity, specificity, interpretability, and resilience to missing data (169).
6) Co-development, education, and support are central to the success of multimodal animal health monitoring, as effective deployment depends on aligning technologies with farmer needs and decision-making contexts. Co-development with farmers ensures practical relevance, while ongoing education and support enable effective interpretation and use of system outputs (169, 170). Evidence from livestock systems indicates that perceived ease of use and demonstrable economic benefit are stronger drivers of adoption than technical sophistication (152, 160), with stakeholder groups differing in their readiness to adopt new technologies (151).
7) Governance frameworks are equally important in shaping adoption outcomes. Reviews of digital agriculture highlight the risk of a widening digital divide, whereby well-capitalized operations are better positioned to adopt advanced monitoring technologies than smaller or resource-constrained producers (161). In extensive systems, particularly in low- and middle-income contexts, ensuring equitable access to new tools and data is therefore both a technical and institutional challenge. Future research should consider not only what multimodal monitoring systems can detect, but also how benefits are distributed and under what governance arrangements.

In the long term, the success of multimodal monitoring will depend on the ability to design systems that are both scientifically robust and practically deployable. Integration must move beyond algorithmic performance to consider adaptability, resilience, and long-term sustainability. By aligning technological innovation with the realities of extensive livestock production, the next generation of monitoring systems can support earlier disease detection, improved animal health and welfare, enhanced productivity, and more sustainable farming systems.

## 6 Conclusion

Multimodal monitoring provides a transformative framework for understanding and managing animal health in extensive livestock systems. Integrating these complementary data streams enables a more mechanistic understanding of health outcomes and resilience under field conditions. This multidimensional perspective offers opportunities for earlier detection of disease, improved welfare outcomes, and more targeted management strategies.

The challenge now is to move beyond proof-of-concept studies toward systems that are reliable, scalable, and capable of operating under the realities of extensive production systems. Advances in data integration, edge computing, federated learning, digital twins, and agentic AI provide promising pathways to achieve this goal.
